# Publisher Correction: A programmable hybrid digital chemical information processor based on the Belousov-Zhabotinsky reaction

**DOI:** 10.1038/s41467-024-46859-8

**Published:** 2024-03-18

**Authors:** Abhishek Sharma, Marcus Tze-Kiat Ng, Juan Manuel Parrilla Gutierrez, Yibin Jiang, Leroy Cronin

**Affiliations:** School of Chemistry, The University of Glasgow, University Avenue, Glasgow, G12 8QQ UK

**Keywords:** Chemical physics, Information technology, Information theory and computation

Correction to: *Nature Communications* 10.1038/s41467-024-45896-7, published online 5 March 2024

The original version of the Peer Review File associated with this Article was updated shortly after publication to remove some comments corresponding to a different article.

