# Supplementary material for: A programmable hybrid digital chemical information processor based on the Belousov-Zhabotinsky reaction
Source: Nat Commun. 2024 Mar 5;15:1984. doi: 10.1038/s41467-024-45896-7 (PMC10915172; doi:10.1038/s41467-024-45896-7)
Supplement: Supplementary file 3 — Description of Additional Supplementary Files [file 41467_2024_45896_MOESM3_ESM.docx]

Inventory of Supplementary Information

Supplementary Video 1:

Description of the automated experimental platform.

Supplementary Video 2:

SM2 Hydrodynamic coupling between nearest neighbouring cells monitor using ink as a tracer.

Supplementary Video 3:

SM3 Implementation of elementary cellular automata rule 30 using a dynamic feedback loop.

Supplementary Video 4:

SM4 Experimental implementation of 2D-CCA.

Supplementary Video 5:

SM5 Simulation of Chemits on 100 ×100 array with different initial conditions (number of Chemits).

Supplementary Video 6:

SM6 Solving four number partitioning problem demonstrating chemical decision making.
